# The impact of coexisting diabetes mellitus on clinical outcomes in patients with idiopathic membranous nephropathy: a retrospective observational study

**DOI:** 10.1186/s12882-020-01878-7

**Published:** 2020-06-12

**Authors:** Zhiyong Xie, Zhilian Li, Wei Dong, Yuanhan Chen, Ruizhao Li, Yanhua Wu, Huaban Liang, Zhiming Ye, Shuangxin Liu, Wei Shi, Xinling Liang

**Affiliations:** 1 Division of Nephrology, Guangdong Provincial People’s Hospital, Guangdong Academy of Medical Sciences, No.106 Zhongshan Road 2, Guangzhou, 510080 Guangdong China; 2 The Second School of Clinical Medicine, Southern Medical University, Guangzhou, 510515 Guangdong China

**Keywords:** Idiopathic membranous nephropathy, Diabetes mellitus, Clinical outcome

## Abstract

**Background:**

Idiopathic membranous nephropathy (IMN) is frequently coexisted with diabetes mellitus (DM). Few researches investigate clinical outcomes in IMN patients coexisting diabetes mellitus (DM), including remission rates, renal survival and complications. Concurrent DM also pose therapeutic challenges to IMN patients due to the influence of glucocorticoids and immunosuppressant on metabolic disorders. We performed this study to investigate the impact of DM on clinical outcomes in IMN and the influence of therapeutic regime on metabolic parameters in diabetic IMN patients.

**Methods:**

Two hundred and six adult hospitalized patients diagnosed with biopsy-proven IMN were retrospectively studied, including 42 patients coexisted with DM. Clinical outcomes including remission rates, renal outcome and complications were compared between groups. Impact of cyclophosphamide and ciclosporin on metabolism and complications were analyzed in IMN patients coexisting DM.

**Results:**

IMN patients coexisted with DM were presented with advanced age, lower level of eGFR and hemoglobin. Patients coexisted with DM experienced worse renal function deterioration and higher incidence of infection. COX regression analysis showed that DM was an independent risk factor for renal function deterioration in IMN patients. There was no significant difference in remission rates and incidence of venous thromboembolism between two groups. Further exploration on the impact of therapeutic regimens on complications and metabolism showed that cyclophosphamide and ciclosporin had no significant difference in incidence of complications including infection and venous thromboembolism, and posed comparable influences on blood glucose, uric acid and blood lipids in IMN patients coexisted with DM.

**Conclusion:**

Coexisting DM was an independent risk factor for renal function deterioration in IMN patients but did not influence the remission of proteinuria. Glucocorticoids in combination with cyclophosphamide or ciclosporine had similar impact on complications and metabolic index including blood glucose, uric acid and blood lipids in IMN patients coexisted with DM.

## Background

Idiopathic membranous nephropathy (IMN) is the most frequent cause of nephrotic syndrome in adult. Recently, the incidence of IMN have increased rapidly and is second only to IgA nephropathy [1]. With the rising prevalence of diabetes mellitus (DM) [2] and chronic kidney disease (CKD) [3], IMN accompanying DM is frequently seen in the spectrum of chronic glomerular disease. IMN coexisted with DM are increasing significantly in the past decades and IMN was found to be the most common glomerular diseases in the diabetic patients [4–7]. The U. S epidemiological data showed that the proportion of MN was 8.2% in the diabetic patients with nondiabetic renal disease (NDRD) [8], whereas the Chinese data showed the prevalence of MN coexisted with DM was 39.3% [9]. However, there are few researches investigating clinical outcomes in IMN patients coexisting DM, including remission rates, renal survival and complications. Also, coexistence of diabetes may pose therapeutic challenges to IMN patients. Firstly, the doses of glucocorticoids in different therapeutic regimen are diverse. Nephrologists may prefer lower doses of prednisone when making descriptions in diabetic IMN patients. Secondly, common therapeutic regimen for high risk IMN patients including glucocorticoids, ciclosporin and tacrolimus would exert an influence on metabolic disorders including hyperglycemia, hyperuricemia and lipid disorders and would increase complications such as infection and venous thromboembolism [10]. Therefore, the impact of different therapeutic regimen on metabolism and complications should be further explored in IMN patients accompanying DM.

Hence, we performed this retrospective observational study to explore clinical outcomes in IMN patients coexisted with DM and analyze the influence of cyclophosphamide and ciclosporin on metabolic parameters in diabetic IMN patients.

## Methods

### Study participants

Three hundred and twenty-nine patients with biopsy-proven membranous nephropathy (MN) were included in this study in Guangdong Provincial People’s Hospital from September 2015 to November 2017. To perform this study, we defined the inclusion criteria as follows: (1) Patients were more than 18 years old at onset; (2) All patients were followed up for at least 6 months. The exclusion criteria were defined as follows: (1) Secondary membranous nephropathy caused by systemic lupus erythematosus, Sjogren’s syndrome, malignancy, hepatitis B virus infections and medication. (2) Patients coexisting diabetic nephropathy (DN) and other chronic glomerular diseases proven by kidney biopsy. Among 329 patients, there were 6 IMN patients coexisted with DN. Considering massive proteinuria in DN would influence the evaluation of remission in IMN, we eliminated IMN patients accompanying DN. Finally, 206 patients were enrolled in this study, including 42 IMN patients with DM. This study applied a retrospective design and the Clinical Research Ethics Committee of Guangdong Provincial People’s Hospital waived the requirement for informed consent in consideration of the retrospective nature of the study.

### Data collection

The baseline clinical and pathological data of participants were collected manually from electronic medical records. The clinical data included age, sex, blood pressure, proportion of hypertension and glomerular hematuria, serum creatinine, estimated Glomerular Filtration Rate (eGFR), 24-h proteinuria,24-h albuminuria, urine protein creatinine ratio (uPCR), urine albumin creatinine ratio (uACR), serum albumin, hemoglobin, blood uric acid, cholesterol (CHOL), triglyceride (TG), high density lipoprotein (HDL), low density lipoprotein (LDL), glycosylated hemoglobin, type of diabetes, duration of diabetes, proportion of different hypoglycemic therapeutic schemes. MN was diagnosed based on the pathological parameters including light microscopy, immunofluorescence and electron microscopy. The pathological data included Ehrenreich-Churg stage, glomerular sclerosis (global sclerosis and segmental sclerosis), tubular atrophy, interstitial inflammatory cell infiltration, interstitial fibrosis, renal arteriolopathy, average glomerular basement membrane thickness (GBMT), glomerulus antigen deposit for IgG subgroups (IgG1, IgG2, IgG3, and IgG4), IgA, IgM, C3, C1q. Detection of glomerular phospholipase A2 receptor (PLA2R) antigen were performed in renal biopsy specimens of all participants. Detection of glomerular Thrombospondin type I domain containing 7A (THSD7A) antigen had been performed since June 1st 2017 in our center and 47 consecutive IMN patients were available for detection of glomerular THSD7A antigen staining in this retrospective observational study.

### Therapy protocol

Secondary causes of membranous nephropathy were clinically ruled out in all MN patients and therapeutic regimen was made by two or more experienced experts according to clinical experience and guideline [11]. Therapy protocols of IMN included immunosuppressive regimens and supportive therapy. Immunosuppressive regimens included glucocorticoids plus cyclophosphamide, glucocorticoids plus ciclosporin, glucocorticoids plus tacrolimus, glucocorticoids plus mycophenolate mofetil and glucocorticoids plus rituximab. Supportive therapy mainly included renin-angiotensin system inhibitor (RASI), diuretics, anticoagulants and lipid-lowering drugs. Therapeutic regimens for DM included oral hypoglycemic drugs, insulin and lifestyle management. Lifestyle management included diabetes self-management education and support, medical nutrition therapy, physical activity, smoking cessation counseling, and psychosocial care without using medicine [12].

### Definitions of clinical indicators

Hypertension was defined as systolic blood pressure ≥ 140 mmHg or diastolic blood pressure ≥ 90 mmHg or the use of anti-hypertensive drugs. Glomerular hematuria was defined as urinary red blood cell (RBC) greater than 8000/mL and dysmorphic RBC accounted for greater than 75% of the total number of RBC after eliminating urinary infections, malformations and stone of urinary tract [13]. DM was diagnosed with fasting blood glucose ≥7.0 mmol/L, 2 h-blood glucose ≥11.1 mmol/L during an oral glucose tolerance test (OGTT), or a previous diagnosis of DM on the basis of the diagnostic criteria by the World Health Organization in 1999. Steroid diabetes was defined by a new-onset diagnosis of DM in IMN inpatients with previous medication history of steroid before admission or a previous diagnosis of steroid diabetes. Complete remission was defined as urinary protein excretion< 0.3 g/d (uPCR< 300 mg/gCr or < 30 mg/mmol), accompanied by a normal serum albumin and serum creatine. Partial remission was defined as urinary protein excretion < 3.5 g/d (uPCR < 3500 mg/gCr or < 350 mg/mmol) and a 50% or greater reduction from peak values, accompanied by an improvement or normalization of serum albumin and stable serum creatine [11]. The presence of complete remission or partial remission were defined as remission. eGFR was calculated according to Chronic Kidney Disease-Epidemiology Collaboration (CKD-EPI) formula [14]. The renal outcome was defined as the composite of end-point of renal function deterioration. The end-point of renal function deterioration was defined as a decrease in the eGFR to 30% of the baseline level or progression to end-stage renal disease (ESRD) during the follow-up [15].

### Statistical analyses

All data were analyzed using *SPSS* statistical software for Windows, version 23.0 (*SPSS, Inc., Chicago, IL, USA*). The measurement data accorded with normal distribution were expressed as the mean ± SD and differences between two groups were compared using the t tests. The measurement data disaccorded with normal distribution were expressed as medians (25th, 75th percentiles) and groups were compared using Mann-Whitney U test. Categorical data were expressed as percentages and differences between two groups were compared using the χ^2^ test or Fisher’s exact test. Kaplan-Meier survival analysis was performed to compare remission rates and renal function outcomes between two groups, and log-rank test was used to evaluate significance of differences. Cox regression analysis was used to adjust for other factors associated with survival and baseline variables with significant difference. Differences were considered to be statistically significant when the *P* value was less than 0.05.

## Results

### Clinicopathological characteristics of IMN patients accompanying DM

Among 206 biopsy-proven IMN patients, 42 (20.4%) IMN patients coexisted with DM. Type 2 Diabetes accounted for 73.8% of subjects investigated while 2.4% was Type 1 Diabetes and 23.8% was steroid diabetes. Almost two thirds were newly diagnosed DM. At the beginning of immunosuppressive therapy, 27 patients received oral hypoglycemic drugs, 1 patient required insulin introduction while 14 patients required lifestyle management. Compared with non-DM patients, the patients with DM exhibited older age, lower eGFR and hemoglobin level, higher glycosylated hemoglobin while other clinical characteristics had no significant difference (Table 1). Light microscopy, immunofluorescence and electron microscopy examinations showed that there were no significant difference in Ehrenreich-Churg stage, glomerular sclerosis (global sclerosis and segmental sclerosis), tubular atrophy, interstitial inflammatory cell infiltration, interstitial fibrosis, renal arteriolopathy, average GBMT, glomerulus antigen deposit for IgG (IgG1, IgG2, IgG3, and IgG4), IgA, IgM, C3, C1q, PLA2R and THSD7A between two groups (Table 2).

**Table 1:** Baseline clinical characteristics in IMN patients with and without DM

Clinical characteristics	Non-DM (***n*** = 164)	DM (***n*** = 42)	***P***-value
**Age (years)**	48 ± 15	60 ± 9	< 0.001
**male (n, %)**	94(57.3)	21 (50.0)	0.394
**SBP (mmHg)**	134 ± 20	137 ± 21	0.505
**DBP (mmHg)**	82 ± 12	78 ± 11	0.062
**Hypertension (n, %)**	71 (43.3)	17 (40.5)	0.742
**Glomerular hematuria (n, %)**	15 (9.1)	3 (7.1)	0.682
**eGFR (ml/min/1.73m**^**2**^**)**	91.5 ± 21.9	76.1 ± 27.1	0.001
**Serum creatinine (μmol/L)**	84 ± 45	96 ± 55	0.134
**Proteinuria (g/24 h)**	5.5 ± 3.8	7.2 ± 6.0	0.084
**Albuminuria (g/24 h)**	3.1 ± 2.1	3.4 ± 2.4	0.522
**uPCR (mg/gCr)**	4.4 ± 3.6	5.9 ± 4.9	0.074
**uACR (mg/gCr)**	2.4 ± 1.9	2.8 ± 2.2	0.173
**Serum albumin (g/L)**	22.4 ± 7.4	22.4 ± 5.7	0.943
**Hemoglobin (g/L)**	129.3 ± 20.2	120.3 ± 21.6	0.012
**Blood uric acid (μmol/L)**	427.2 ± 120.5	402.8 ± 103.8	0.232
**Cholesterol (mmol/L)**	8.7 ± 3.0	8.2 ± 2.5	0.349
**Triglyceride (mmol/L)**	3.1 ± 2.2	3.3 ± 2.1	0.588
**HDL (mmol/L)**	1.5 ± 0.5	1.5 ± 0.5	0.652
**LDL (mmol/L)**	5.5 ± 2.0	5.1 ± 1.7	0.233
**Glycosylated hemoglobin (%)**	5.6 ± 0.5	6.5 ± 1.0	< 0.001
**Type of Diabetes (n, %)**			
**Type 1 Diabetes**	–	1 (2.4)	–
**Type 2 Diabetes**	–	31 (97.6)	–
**Steroid diabetes**	–	10 (23.8)	
**Duration of diabetes (n, %)**			
**New-onset diabetes**	–	27 (64.3)	–
**Previous diabetes**	–	15 (35.7)	–
**Therapeutic regimen for DM (n, %)**			
**Oral hypoglycemic drugs**	–	27 (64.3)	–
**Insulin**	–	1 (2.4)	–
**Lifestyle management**	–	14 (33.3)	–

Data were expressed as mean ± SD (standard deviation), medians (interquartile range), or numbers (%)

*DM* diabetes mellitus, *SBP* systolic blood pressure, *DBP* diastolic blood pressure, *eGFR* estimated glomerular filtration rate, *uPCR* urine protein creatinine ratio, *uACR* urine albumin creatinine ratio, *HDL* high density lipoprotein, *LDL* low density lipoprotein

**Table 2:** Baseline pathological characteristics in IMN patients with and without DM

Pathological characteristics	Non-DM (***n*** = 164)	DM (***n*** = 42)	***P***-value
**Ehrenreich-Churg stage**			0.578
**MN I + II (n, %)**	124 (75.6)	30 (71.4)	
**MN III + IV (n, %)**	40 (24.4)	12 (28.6)	
**Glomerular sclerosis (n, %)**			
**Global sclerosis**	39 (23.8)	9 (21.4)	0.748
**Segmental sclerosis**	35 (21.3)	8 (19.4)	0.744
**Tubular atrophy (n, %)**			0.889
**Atrophy area < 25%**	157 (95.7)	40 (95.2)	
**Atrophy area ≥ 25%**	7 (4.3)	2 (4.8)	
**Interstitial ICI (n, %)**	143 (87.2)	38 (90.5)	0.561
**Interstitial fibrosis (n, %)**	54 (32.9)	14 (33.3)	0.960
**Renal arteriopathy (n, %)**	131 (79.9)	33 (78.6)	0.851
**IgG subgroup deposit (n, %)**			
**IgG1 (n, %)**	133 (81.1)	35 (83.3)	0.739
**IgG2 (n, %)**	103 (62.8)	26 (61.9)	0.914
**IgG3 (n, %)**	73 (44.5)	18 (42.9)	0.847
**IgG4 (n, %)**	155 (94.5)	39 (92.9)	0.683
**IgA deposit (n, %)**	4 (2.4)	2 (4.8)	0.424
**IgM deposit (n, %)**	137 (83.5)	34 (81.0)	0.691
**C3 deposit (n, %)**	158 (96.3)	42 (100)	0.350
**C1q deposit (n, %)**	5 (3.0)	3 (7.1)	0.220
**Positive PLA2R deposit (n, %)**	127 (77.4)	35 (83.3)	0.406
**Positive THSD7A deposit (n, %)**^**a**^	0 (0)	0 (0)	–
**Average GBMT (nm)**	1440 ± 338	1524 ± 316	0.150

Data were expressed as mean ± SD (standard deviation) or numbers (%)

*DM* diabetes mellitus, *MN* membranous nephropathy, *ICI* inflammatory cell infiltration, *PLA2R* Phospholipase A2 Receptor, *THSD7A* Thrombospondin Type-1 Domain containing 7A, *GBMT* glomerular basement membrane thickness

^a^The detection of glomerular THSD7A antigen staining were available for 47 consecutive IMN patients between June 2017 to November 2017 due to the retrospective nature of the study

### Clinical outcomes and complications in IMN patients with coexisting DM

Among 206 patients enrolled in the study, 152 patients (73.8%) received immunosuppressive therapy, including glucocorticoids plus ciclosporin (46.6%), glucocorticoids plus cyclophosphamide (9.7%), glucocorticoids plus tacrolimus (11.2%) and others. IMN patients coexisted with DM received higher proportion of cyclophosphamide while there was no significant difference in the proportion of other kinds of immunosuppressive therapy between IMN patients with and without coexisting DM. No significant difference was found in the accumulated glucocorticoids dosage between two groups (Table 3).

**Table 3:** Therapeutic regimen in IMN patients with and without coexisting DM

Therapeutic regimen	Total (***n*** = 206)	Non-DM (***n*** = 164)	DM (***n*** = 42)	***P***-value
**Non-Immunosuppressive therapy (n, %)**	54 (26.2)	44 (26.8)	10 (23.8)	0.691
**Immunosuppressive therapy (n, %)**	152 (73.8)	120 (73.2)	32 (76.2)	
**Glucocorticoids dosage (g)**	1.8 (1.8,2.7)	1.8 (1.8,2.7)	1.8 (1.9,3.4)	0.586
**CTX (n, %)**	20 (9.7)	12 (7.3)	8 (19.0)	0.037
**CsA (n, %)**	96 (46.6)	76 (46.4)	20 (47.6)	0.882
**FK506 (n, %)**	23 (11.2)	20 (12.2)	3 (7.2)	0.425
**Other**^a^**(n, %)**	13 (6.3)	12 (7.3)	1 (2.4)	0.474

Data were expressed as numbers (%). All immunosuppressive therapy was combined with glucocorticoids

*DM* diabetes mellitus, *CTX* cyclophosphamide, *CsA* ciclosporin, *FK506* tacrolimus

^a^other therapeutic regimens included mycophenolate mofetil and glucocorticoids, rituximab and glucocorticoids

**Fig. 1:**
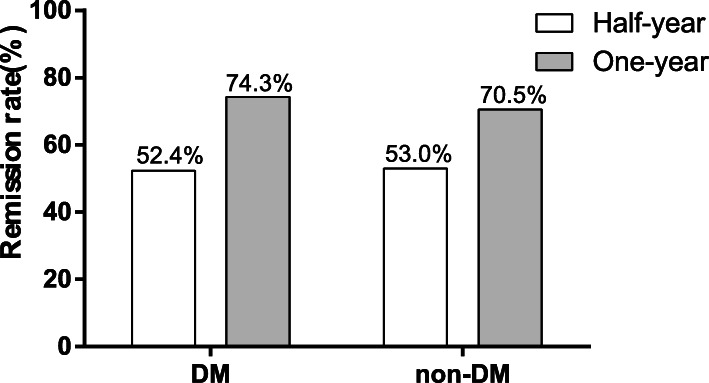
Comparison of remission rates in IMN patients with and without DM. The half-year and one-year remission rates were shown as Bar graph. Differences between two groups were compared using χ^2^ tests

**Fig. 2:**
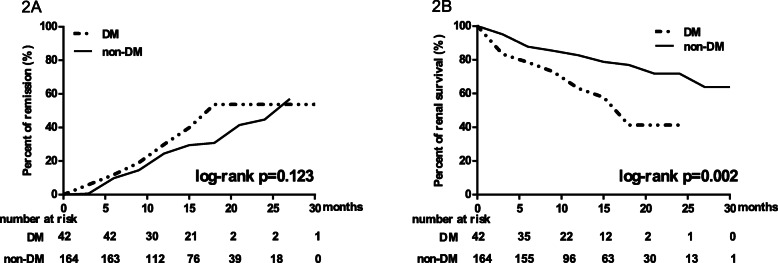
Kaplan-Meier analysis of remission rates and renal function survival in IMN patients with and without DM. **a** Survival curve for reaching remission, including partial remission or complete remission (Log-rank test, *P* = 0.123). **b** Survival curve for reaching > 30% decline of eGFR or doubling of serum creatine or eGFR< 15 ml/min/1.73m^2^ (Log-rank test, *P* = 0.002). Log-rank test was used to evaluate the significance of differences

Remission rates were compared between two groups (Fig. 1). In diabetic and non-diabetic IMN patients, half-year remission rates were respectively 52.4 and 53.0% (χ^2^ = 0.006, *P* = 0.938) while one-year remission rates were respectively 74.3 and 70.5% (χ^2^ = 0.189, *P* = 0.664). There were no statistically significant differences in remission rates between two groups. Kaplan-Meier analysis showed that after a median follow-up of 15 (9,18) months there was no statistically significant difference in remission rates between two groups (Log-rank test, *P* = 0.123) (Fig. 2a).

For renal function outcome, among 42 patients coexisted with DM, 8 patients (19.0%) reached renal end point, including 1 patient (2.4%) progressing to ESRD after a median follow-up duration of 12 (6,15) months. Among 164 patients without coexisting DM, 29 patients (17.7%) reached renal end point, including 2 patients (1.2%) progressing to ESRD after a median follow-up duration of 12 (6,15) months. Kaplan-Meier analysis showed that IMN patients with coexisting DM experienced severer renal function deterioration than those without coexisting DM (Log-rank test, *P* = 0.002) (Fig. 2b). Cox univariate analysis showed that DM increased risk for renal function deterioration (Hazard Ratio: 2.398; 95% CI:1.327 to 4.333; *P* = 0.004). After adjusting for variables with significant differences in Cox univariate analysis, including eGFR, albuminuria, hemoglobin, interstitial fibrosis and therapeutic regimen, DM was still an independent risk factor for renal function deterioration (Hazard Ratio: 1.988; 95% CI: 1.069 to 3.695; *P* = 0.030) (Table 4).

**Table 4:** Factors predicting renal function deterioration in Cox regression models

Variables	Cox univariate analysis		Cox multivariate analysis	
	HR (95% CI)	***p***-value	HR (95% CI)	***p***-value
**DM**	2.398 (1.327,4.333)	0.004	1.988 (1.069,3.695)	0.030
**Age**	1.056 (1.033,1.080)	< 0.001	–	–
**eGFR**	0.987 (0.977,0.996)	0.005	0.991 (0.981,1.002)	0.109
**Albuminuria**	0.953 (0.910,0.998)	0.039	0.964 (0.917,1.014)	0.160
**Hemoglobin**	0.984 (0.972,0.996)	0.010	0.991 (0.977,1.004)	0.185
**Interstitial fibrosis**	1.944 (1.108,3.411)	0.020	1.996 (1.127,3.533)	0.018
**Therapeutic regimen**		0.208		0.182
**Supportive therapy**	Reference	Reference	Reference	Reference
**CTX**	1.951 (0.619,6.151)	0.254	1.034 (0.312,3.424)	0.956
**CsA**	2.364 (1.037,5.390)	0.041	2.201 (0.941,5.149)	0.069
**FK506**	2.214 (0.774,6.330)	0.138	2.124 (0.731,6.173)	0.166
Other*	0.557 (0.068,4.530)	0.584	0.550 (0.067,4.548)	0.579

Considering the existence of collinearity between age and eGFR, we only included eGFR in Cox Multivariate Analysis. The variables were selected in the models in an “enter” manner

*HR* hazard ratio, *CI* Confidence interval, *DM* diabetes mellitus, *eGFR* estimated Glomerular Filtration Rate, *CTX* cyclophosphamide, *CsA* ciclosporin, *FK506* tacrolimus

* including mycophenolate mofetil and glucocorticoids, rituximab and glucocorticoids

**Fig. 3:**
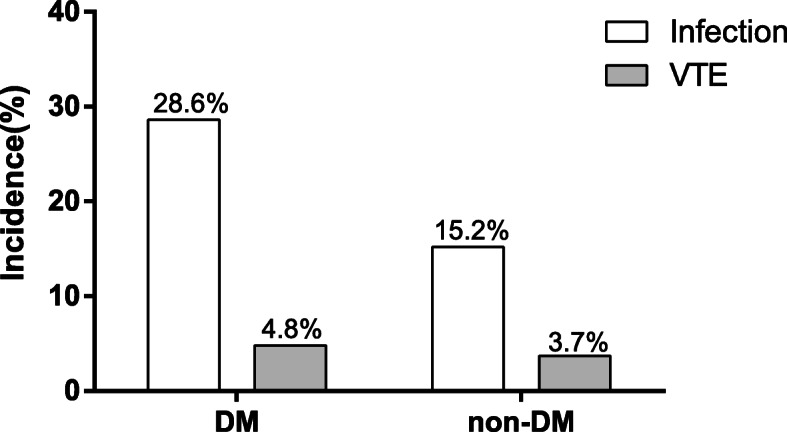
Comparison of complications in IMN patients with and without DM. The incidence of infection and venous thromboembolism were shown as Bar graph. Differences between two groups were compared using χ^2^ tests. VTE: venous thromboembolism

Incidence of complications including infection and venous thromboembolism were compared between IMN patients with and without coexisting DM (Fig. 3). The incidence of infection in patients with DM was higher than that in patients without DM (28.6 and 15.2%, χ^2^ = 4.031, *P* = 0.045) while there was no difference in the incidence of venous thromboembolism between two groups (4.8 and 3.7%, χ^2^ = 0.109, *P* = 0.667).

### Effect of glucocorticoids in combination with cyclophosphamide and ciclosporin on complications and metabolic parameters in IMN patients with coexisting DM

**Fig. 4:**
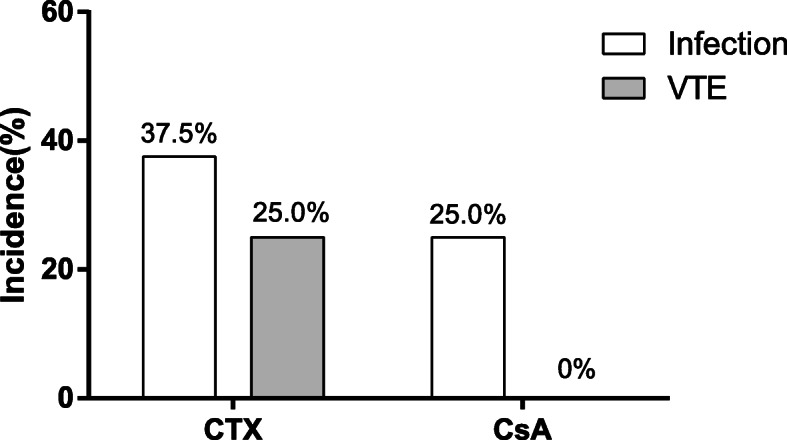
Comparison of complications in CTX and CsA among IMN patients with DM. VTE: venous thromboembolism; CTX: cyclophosphamide; CsA: ciclosporin. The incidence of infection and VTE were shown as Bar graph. Differences between two groups were compared using χ^2^ tests

Only 3 diabetic IMN patients received tacrolimus therapy, thereby we compared the complications including infection and venous thromboembolism between cyclophosphamide and ciclosporin in diabetic IMN patients (Fig. 4). The incidence of infection in patients using cyclophosphamide and ciclosporin were respectively 37.5 and 25.0% (χ^2^ = 0.437, *P* = 0.651) while the incidence of venous thromboembolism was respectively 25.0 and 0% (χ^2^ = 5.385, *P* = 0.074). There was no significant difference in the incidence of infection and venous thromboembolism using cyclophosphamide or ciclosporin in IMN patients with coexisting DM.

Influences on blood glucose, uric acid and blood lipids parameters were further compared between cyclophosphamide and ciclosporin in IMN patients coexisted with DM (Table 5). The accumulative glucocorticoid doses in cyclophosphamide and ciclosporin were respectively (6.2 ± 2.5) g and (2.2 ± 0.9) g, with statistically significant difference. Among IMN patients with coexisting DM, pre-treatment, post-treatment and change value of fasting blood glucose, uric acid and blood lipids including CHOL, TG, HDL and LDL from initial time to the end of follow-up in cyclophosphamide and ciclosporin had no statistically significant difference (*P* < 0.05). Among 8 IMN accompanying DM patients treated with cyclophosphamide, 2 patients under lifestyle management experienced blood glucose fluctuation and oral hypoglycemic drugs were required to improve glycemic control. As for the glycemic control after at least 6 months of ciclosporin in combination of low dose of glucocorticoids, 6 of 20 patients experienced blood glucose fluctuation but the original scheme could achieve improved glycemic control.

**Table 5:** The influence of cyclophosphamide or ciclosporin on metabolism in IMN patients with coexisting DM

	DM		
	CTX	CsA	***p-***value
Accumulative GC dose (g)	6.2 ± 2.5	2.2 ± 0.9	< 0.001
**Fasting blood glucose**			
Pre-treatment FBG (mmol/L)	9.6 ± 3.4	8.4 ± 4.5	0.516
Post-treatment FBG (mmol/L)	5.8 ± 1.5	5.9 ± 1.5	0.826
Changes of FBG (mmol/L)	−2.8(−5.0, −1.0)	−1.2(−3.6, 0.2)	0.110
Goal Attainment Rates of FBG (%)	62.5%	70%	0.701
**Uric acid**			
Pre-treatment uric acid (μmol/L)	376.5 ± 63.9	428.2 ± 123.4	0.272
Post-treatment uric acid (μmol/L)	351.1 ± 67.5	418.7 ± 142.1	0.213
Changes of uric acid (μmol/L)	−12.5(−101.3,56.5)	8.5(− 107.9,75.8)	0.672
Goal Attainment Rates of uric acid (%)	87.5%	70%	0.334
**Cholesterol**			
Pre-treatment CHOL (mmol/L)	7.2 ± 2.4	8.7 ± 3.0	0.196
Post-treatment CHOL (mmol/L)	4.6 ± 0.8	4.6 ± 2.8	0.929
Changes of CHOL (mmol/L)	−2.0(−5.6,0.1)	−3.5(−6.2, −1.2)	0.940
Goal Attainment Rates of CHOL (%)	87.5%	55%	0.105
**Triglyceride**			
Pre-treatment TG (mmol/L)	3.2 ± 2.7	3.3 ± 1.7	0.898
Post-treatment TG (mmol/L)	1.8 ± 0.8	2.2 ± 1.0	0.391
Changes of TG (mmol/L)	−0.9(−2.3, −0.3)	−0.6(− 2.5,0)	0.709
Goal Attainment Rates of TG (%)	50%	40%	0.629
**High density lipoprotein**			
Pre-treatment HDL (mmol/L)	1.5 ± 0.6	1.5 ± 0.4	0.982
Post-treatment HDL (mmol/L)	1.4 ± 0.5	1.5 ± 0.6	0.617
Changes of HDL (mmol/L)	−0.2(−0.3,0)	0(−0.3,0)	0.533
Goal Attainment Rates of HDL (%)	50%	60%	0.629
**Low density lipoprotein**			
Pre-treatment LDL (mmol/L)	4.4 ± 1.6	5.4 ± 2.0	0.221
Post-treatment LDL (mmol/L)	2.7 ± 0.7	2.3 ± 2.0	0.634
Changes of LDL (mmol/L)	−1.7(−3.5,0.2)	−3.7(−4.6, −1.0)	0.784
Goal Attainment Rates of LDL (%)	100%	60%	0.063

Data were expressed as mean ± SD (standard deviation), medians (interquartile range), or percentage (%)

*GC* glucocorticoid, *FBG* fasting blood glucose, *CHOL* cholesterol, *TG* triglyceride, *HDL* high density lipoprotein, *LDL* low density lipoprotein, *CTX* cyclophosphamide, *CsA* ciclosporin

## Discussion

DM is increasing significantly in recent year in China [16]. With the high incidence of DM and the surging prevalence of CKD [17], chronic glomerular disease coexisted with DM is not uncommon. Recently, incidence of IMN had also increased significantly and epidemiological studies based on the spectrum of glomerular diseases had showed that IMN was the most common primary glomerular disease in diabetic patients [4–7, 9] with incidence ranging from 20.9 to 39.3%. Epidemiological data of glomerular diseases in our center showed that the proportion of DM in patients performed renal biopsy was 8.9% (537/6044) from January 2001 to June 2018, and IMN was found to be the most common primary glomerular disease coexisted with DM, with the proportion of 20.9% (114/537). Coexisting DM would lead to renal hemodynamic changes, increase in the amount of glucose filtered through the glomerular filtration barrier, overactive of renin angiotensin aldosterone system [18, 19], which may pose influence on proteinuria remission and renal function deterioration. However, few studies [20] have ever investigated the influence of DM on clinical outcomes in IMN. Additionally, DM is associated with metabolic disorders and an increased risk of infection. Whether coexistence of DM would pose great influence on metabolism in IMN patients still remains unclear, especially after using diverse immunosuppressants and different doses of glucocorticoid. Therefore, the present study was designed to investigate the impact of coexisting DM on clinical outcomes including remission rates, renal outcome and complications in IMN patients. At the same time, we carried out this study to explore the influence of cyclophosphamide and ciclosporin on complication and metabolism including blood glucose, uric acid and blood lipids in IMN patients accompanying DM.

In this study, IMN patients coexisted with DM were significantly older and presented with lower level of eGFR and hemoglobin compared with those without DM, which is consistent with previous reports [16, 21, 22]. In term of renal outcome, DM was an independent risk factor for renal function decline, even after adjusting for potential confounders, including the baseline parameters with significant statistical differences. Several reasons existed for renal function deterioration in IMN patients coexisting with DM. First, hyperglycemia exacerbates renal ischemia-reperfusion injury through an increased inflammatory response and aggravated the degree of renal tubular apoptosis and damage [23]. Second, hyperglycemia would increase the production of angiotensin II, induce endothelial dysfunction and lead to renal hemodynamic changes, including increased intraglomerular capillary pressure and glomerular hyperfiltration [24]. Third, the coexistence of proteinuria would accelerate vascular ageing and endothelial and tubular dysfunction, ultimately leading to renal ischemia and accelerated renal function progression [19]. However, we had found that there was no difference in the half-year and one-year remission rates between IMN patients with and without DM, which indicated that DM may not pose influence on proteinuria as disease had not superimposed on DN.

As for complications in IMN patients coexisted with DM, the incidence of infections in diabetic IMN patients was higher than patients without DM. Patients with DM are more prone to suffering from infections owing to hyperglycemia is favorable circumstance for the growth and reproduction of pathogenic bacteria. In addition, diabetic patients represented lower immunity on account of reduction of immunoglobulin synthesis. Previous studies had indicated that the incidences of infections fluctuated from 9.7 to 50% [25–29] after using cyclophosphamide while ranged from 4.3 to 21.6% with ciclosporin [29–32] in IMN patients. Thromboembolic events were another common and recognized complication in patients with the nephrotic syndrome [33], especially in IMN. Previous studies had showed that the incidence of thrombosis events fluctuated between 14.3 and 34.2% after using cyclophosphamide while ranged between 10.5 and 17.2% with ciclosporin [26, 34] in IMN patients. However, in our study we found no difference in incidence of infection and venous thromboembolism in diabetic IMN patients after using cyclophosphamide or ciclosporin.

Classic therapeutic regimen recommended by guidelines for IMN may lead to metabolic disorders including hyperglycemia, hyperuricemia and lipid disorders. High-dose glucocorticoid [35] applied in cyclophosphamide regimens or ciclosporin [36] all would pose significant influence on metabolism. Higher proportion of new-onset DM was observed in IMN patients, which would pose a great challenge to glycemic management for IMN patients using glucocorticoids and immunosuppressant. Diabetic patients are often accompanied with metabolic disturbance, which exert great challenge to the control of blood glucose, uric acid and blood lipids. There is no published literature examining which therapeutic regimens pose severer influence on metabolism parameter in IMN patients with diabetes. Therefore, the difference of therapeutic medications on metabolism in IMN patients, especially IMN superimposed on DM, should warrant the attention of physicians. In this study, we had indicated that changes of blood glucose, uric acid and blood lipids after using cyclophosphamide or ciclosporin were comparable in diabetic IMN patients in long-term follow-up. Since metabolic disorders associated with cyclophosphamide are rarely seen, we may come to a conclusion that high-dose glucocorticoid in the therapeutic regime of cyclophosphamide and ciclosporin have comparable effects on metabolism. Glucocorticoid could increase insulin resistance and the diabetogenic effects of glucocorticoid may be dose dependent. Hjelmesaeth et al. [37] had found that tapering off glucocorticoid but not ciclosporin could significantly improves glucose tolerance during the first year after transplantation. Therefore, the impact of decreasing in the dosage of glucocorticoid and ciclosporin during the treatment periodicity of IMN still deserve further investigation. Although some IMN patients using therapeutic regime of cyclophosphamide or ciclosporin may experience blood glucose fluctuation, oral hypoglycemic drugs or insulin introduction could achieve improved glycemic control. Seldom immunosuppression withdrawal was found in IMN patient due to treatment-related metabolic disorder. Through using medication or lifestyle management, we found the parameters of blood glucose, uric acid and blood lipids at the end of follow-up were better than metabolize situation at onset.

The strength of this study mainly discussed clinical outcomes in IMN patients coexisted with DM, including remission of proteinuria, renal outcome and complications, which would provide some suggestions for the management and prognosis evaluation in IMN patients coexisted with DM. Further investigation on metabolism would also provide some useful information for treatment options in diabetic IMN patients. However, our study still had some limitations. Nowadays, the management of MN had entered an era of PLA2R [38]. With higher specificity and sensitivity, PLA2R had been used to assess disease activity, clinical outcome and risk of collapse in IMN [39–41]. Due to the retrospective nature of the study, the measurements of serum anti-PLA2R antibody were not available for the enrolled IMN patients at that time. However, detection of glomerular PLA2R antigen were performed in renal biopsy specimens of all participants in this study, which was useful for the diagnosis of IMN. Additionally, small sample sizes enrolled from a single center impose restrictions on its generality and lead to inevitable selection bias. Finally, the median duration of the follow-up was 12 months. Longer follow-up duration was required to explore the long-term impact of coexisting DM on clinical outcomes in IMN patients.

## Conclusions

Coexisting DM was an independent risk factor for renal function deterioration in IMN patients but did not influence the remission of proteinuria. Glucocorticoids in combination with cyclophosphamide or ciclosporine had similar impact on complications and metabolic index including blood glucose, uric acid and blood lipids, which would be conducive to therapeutic options in diabetic IMN patients.

## Supplementary information

**Additional file 1.**

**Additional file 2.**

**Additional file 3.**

**Additional file 4.**

**Additional file 5.**

**Additional file 6.**

## Data Availability

The datasets used and/or analysed during the current study available from the corresponding author on reasonable request.

## Abbreviations

IMN: Idiopathic membranous nephropathy
DM: Diabetes mellitus
CKD: Chronic kidney disease
NDRD: Nondiabetic renal disease
MN: Membranous nephropathy
DN: Diabetic nephropathy
eGFR: Estimated Glomerular Filtration Rate
uPCR: Urine protein creatinine ratio
uACR: Urine albumin creatinine ratio
CHOL: Cholesterol
TG: Triglyceride
HDL: High density lipoprotein
LDL: Low density lipoprotein
GBMT: Glomerular basement membrane thickness
PLA2R: Phospholipase A2 receptor
THSD7A: Thrombospondin type I domain containing 7A
RASI: Renin-angiotensin system inhibitor
RBC: Red blood cell
OGTT: Oral glucose tolerance test
CKD-EPI: Chronic Kidney Disease-Epidemiology Collaboration
ESRD: End-stage renal disease
VTE: Venous thromboembolism
CTX: Cyclophosphamide
CsA: Ciclosporin
