# Supplementary material for: The impact of coexisting diabetes mellitus on clinical outcomes in patients with idiopathic membranous nephropathy: a retrospective observational study
Source: BMC Nephrol. 2020 Jun 12;21:224. doi: 10.1186/s12882-020-01878-7 (PMC7291707; doi:10.1186/s12882-020-01878-7)
Supplement: Supplementary file 4 — Additional file 4. [file 12882_2020_1878_MOESM4_ESM.pdf]

广东省人民医院-广州金域医学检验中心

本报告单经过电子签名认证

病理诊断报告书

标本条码： 0125423882
病人姓名：
性　别：　女
年　龄：　74　岁
项目名称：　免疫荧光8项
送检材料：　肾脏组织
临床诊断：　慢性肾炎综合征

医　院：　广东省人民医院-肾内科
科　室：　肾内科一区
房/床号：　64床
接收时间：　2016-09-13 23:22:52

病理号：　KB1623377
门诊/住院号：P559714
申请医生：
医生电话：　83827812-61421
患者电话：

大体描述:

1：中性甲醛固定的条索状灰白色组织一条，长约（0.8cm）取一盒全用作光镜检查；
2：荧光保存液固定的条索状灰白色组织一条，长约（0.3cm）取一盒全用作免疫荧光检查；
3：中性戊二醛固定的条索状灰黄组织一条，长约（0.1cm）取一盒全用作电镜检查。

镜下描述（主要病变）：

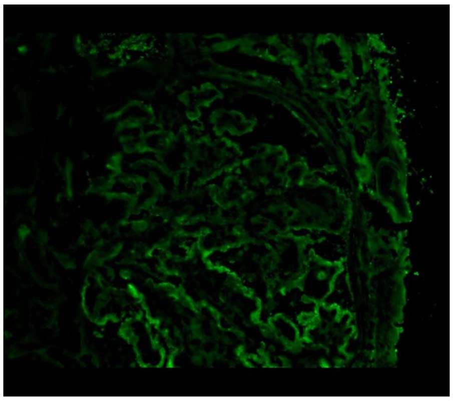

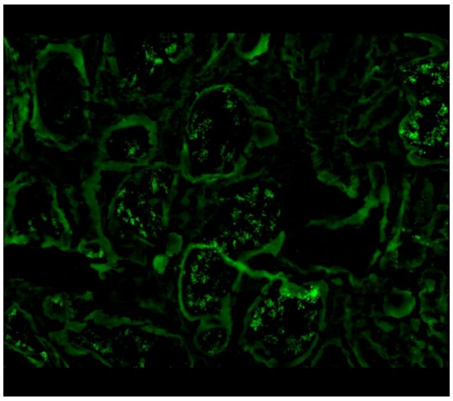

诊断意见:

免疫荧光：IgG1：++，IgG2：阴性，IgG3：阴性，IgG4：++，PLA2R：+，κ：+，λ：+，AA：阴性，HBsAg：阴性，HBcAg：阴性，HBeAg：阴性，Fib：阴性，ALB：可见肾小管重吸收小滴，C4d：阴性。

本检测仅对来样负责。如果对结果有疑义，请在收到结果后7个工作日内与我们联系，多谢合作！

报告医师：

查对技师：

报告日期：　2016-09-17 09:01:01

广东省人民医院-广州金域医学检验中心

本报告单经过电子签名认证

肾活检病理检查电镜报告书

标本条码：	0125423882	医　院：	广东省人民医院-肾内科		
病人姓名：		科　室：	肾内科一区	病理号：	KB1623377
性　别：	女	房/床号：	64床	门诊/住院号	P559714
年　龄：	74　岁	接收日期：	2016-09-13 23:22:52	申请医生：	
项目名称：	普通透射电镜检查与诊断			医生电话：	83827812-61421
送检材料：	肾脏组织			患者电话：	
临床诊断：	慢性肾炎综合征			医院标识：	

大体描述:

1：中性甲醛固定的条索状灰白色组织一条，长约（0.8cm）取一盒全用作光镜检查；
2：荧光保存液固定的条索状灰白色组织一条，长约（0.3cm）取一盒全用作免疫荧光检查；
3：中性戊二醛固定的条索状灰黄组织一条，长约（0.1cm）取一盒全用作电镜检查。

电镜描述:

肾小球：镜下检测到2个肾小球，其中1个肾小球硬化，另1个肾小球毛细血管内皮细胞明显空泡变性，个别管腔内可见红细胞聚集，无明显内皮细胞增生，毛细血管襻开放。肾小囊壁层无明显增厚，壁层细胞空泡变性，无明显增生。
基底膜：弥漫均质增厚，厚度达2000nm，基底膜内见多量电子致密物沉积伴少量吸收。
脏层上皮细胞：上皮细胞肿胀，空泡变性。足突弥漫融合，上皮下见电子致密物沉积。
系膜区：系膜细胞和基质增生，未见电子致密物沉积。
肾小管-间质：肾小管上皮细胞空泡变性，少数肾小管萎缩。肾间质少量炎症细胞浸润。
肾间质血管：个别毛细血管管腔内见红细胞聚集。小动脉管壁增厚。

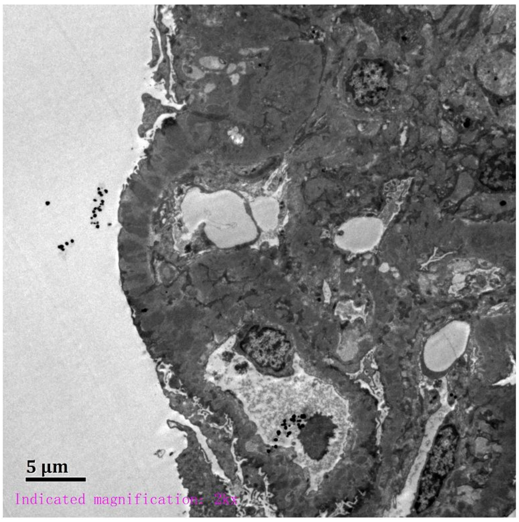

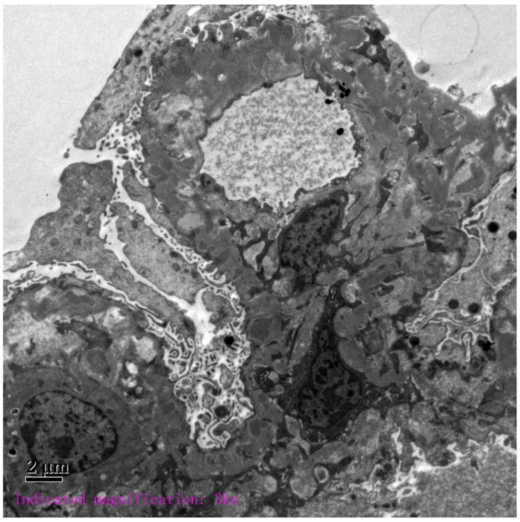

电镜诊断或印象:

综合光镜、免疫荧光及电镜检查：
符合III期膜性肾病。
注：此病例此次肾活检光镜下可见系膜基质明显增生，电镜下基底膜均质增厚，结合临床，考虑为膜性肾病合并糖尿病肾病。

本检测仅对来样负责。如果对结果有疑义，请在收到结果后7个工作日内与我们联系，多谢合作！　　报告医师：

The manifestation of light microscope,immunofluorescence and electron microscope supported the diagnosis of membranous nephropathy stage III.
Mesangial matrix were obviously proliferative under light microscope and the basement membrane was homogeneously thickened under electron microscopy, which supported the diagnosis of coexisting diabetic nephropathy in accordance with clinical manifestations.

肾活检病理检查报告书

标本条码：	0125423882	医　院：	广东省人民医院-肾内科		
病人姓名：		科　室：	肾内科一区	病理号：	KB1623377
性　别：	女	房/床号：	64床	门诊/住院号	P559714
年　龄：	74　岁	接收日期：	2016-09-13 23:22:52	申请医生：	
项目名称：	常规肾脏病理检查			医生电话：	83827812-61421
送检材料：	肾脏组织			患者电话：	
临床诊断：	慢性肾炎综合征				

大体描述:

1：中性甲醛固定的条索状灰白色组织一条，长约（0.8cm）取一盒全用作光镜检查；
2：荧光保存液固定的条索状灰白色组织一条，长约（0.3cm）取一盒全用作免疫荧光检查；
3：中性戊二醛固定的条索状灰黄组织一条，长约（0.1cm）取一盒全用作电镜检查。

光镜描述

送检肾穿刺组织常规做HE、PAS、PASM、Masson染色，包括肾皮质及髓质，可见14个肾小球，其中4个肾小球球性硬化。
其余肾小球系膜细胞和基质中-重度增生，以基质增生更为明显，毛细血管襻开放，外观显僵硬，基底膜增厚，可见较多钉突样结构，上皮下可见嗜复红蛋白沉积，未见纤维素样坏死，未见白金耳样结构，壁层上皮细胞无增生，未见新月体形成，可见1个肾小球球囊周纤维化，其内毛细血管襻缺血、皱缩。
肾小管上皮细胞空泡及颗粒变性，灶状萎缩（萎缩面积约15%），肾间质灶状炎症细胞浸润伴纤维化，小动脉管壁增厚，管腔狭窄。
免疫荧光：IgG1：++，IgG2：阴性，IgG3：阴性，IgG4：++，PLA2R：+，κ：+，λ：+，AA：阴性，HBsAg：阴性，HBcAg：阴性，HBeAg：阴性，Fib：阴性，ALB：可见肾小管重吸收小滴，C4d：阴性。
特殊染色：刚果红：阴性，氧化刚果红：阴性，油红“O”：阴性。

免疫荧光:　肾小球数 6 个

抗体种类:

IgG: +++　　IgM: +　　IgA: 阴性　　C3:　+　　C1q:　阴性

沉积部位: 弥漫、球性、毛细血管襻

沉积方式: 细颗粒状

病理诊断:

综合光镜、免疫荧光及电镜检查：
符合III期膜性肾病。
注：此病例此次肾活检光镜下可见系膜基质明显增生，电镜下基底膜均质增厚，结合临床，考虑为膜性肾病合并糖尿病肾病。

本检测仅对来样负责。如果对结果有疑义，请在收到结果后7个工作日内与我们联系，多谢合作！

报告医师：

[illegible]

The manifestation of light microscope,immunofluorescence and electron microscope supported the diagnosis of membranous nephropathy stage III.
Mesangial matrix were obviously proliferative under light microscope and the basement membrane was homogeneously thickened under electron microscopy, which supported the diagnosis of coexisting diabetic nephropathy in accordance with clinical manifestations.

广东省人民医院-广州金域医学检验中心

本报告单经过电子签名认证

肾活检病理检查报告书图片页

标本条码：	0125423882	医　院：	广东省人民医院-肾内科	病理号：	KB1623377
病人姓名：				门诊/住院号	P559714
性　别：	女	科　室：	肾内科一区	房/床号：	64床
年　龄：	74　岁	接收日期：	2016-09-13 23:22:52	申请医生：	
项目名称：	常规肾脏病理检查			医生电话：	83827812-61421
送检材料：	肾脏组织			患者电话：	
临床诊断：	慢性肾炎综合征				

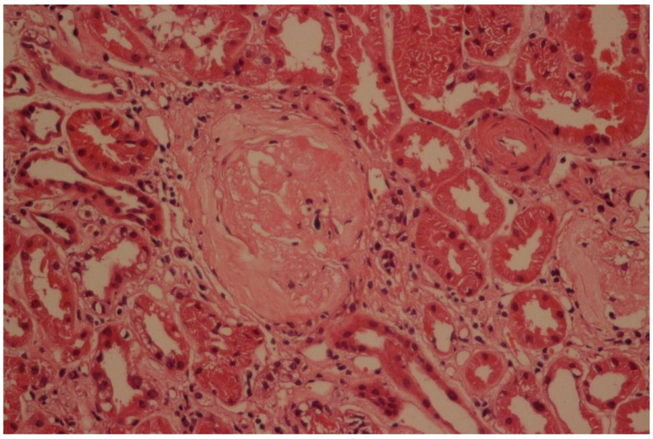

HE见硬化的肾小球

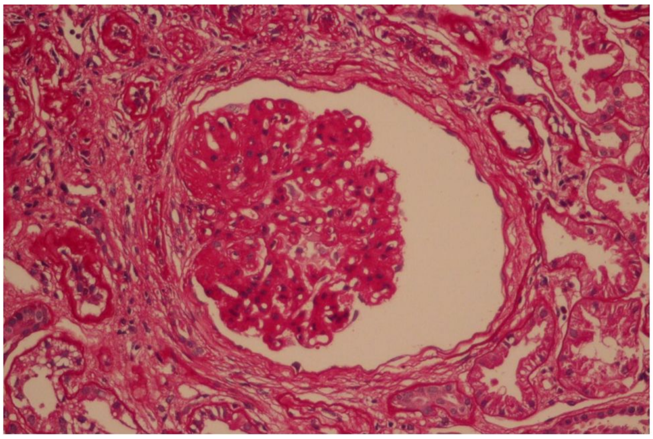

PAS见球囊周纤维化的肾小球

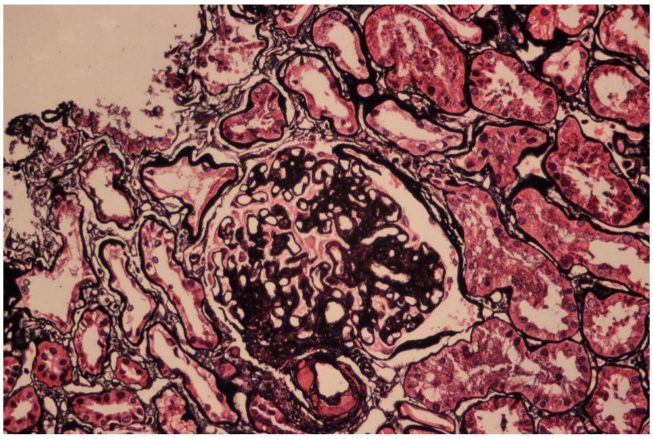

PASM见基底膜增厚，可见较多钉突样结构

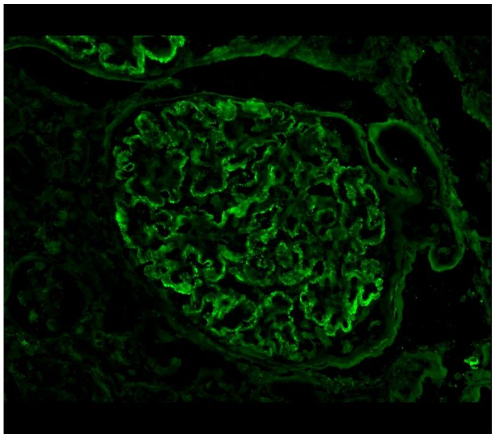

免疫荧光见IgG沉积

本检测为科研检测且仅对来样负责。如果对结果有疑义，请在收到结果后7个工作日内与我们联系，多谢合作！ 报告医师：

主检实验室：肾脏常规岗位

GZ16811413591723

报告日期：　2016-09-17 09:00:45
