# Supplementary material for: The impact of coexisting diabetes mellitus on clinical outcomes in patients with idiopathic membranous nephropathy: a retrospective observational study
Source: BMC Nephrol. 2020 Jun 12;21:224. doi: 10.1186/s12882-020-01878-7 (PMC7291707; doi:10.1186/s12882-020-01878-7)
Supplement: Supplementary file 5 — Additional file 5. [file 12882_2020_1878_MOESM5_ESM.pdf]

病理诊断报告书

标本条码：	0137126028	医　院：	广东省人民医院-肾内科		
病人姓名：		科　室：	肾内科一区	病理号：	KB1737358
性　别：	男	房/床号：	29床	门诊/住院号:	P771218
年　龄：	76　岁	接收时间：	2017-12-13 16:16:09	申请医生：	
项目名称：	免疫荧光15项			医生电话：	83827812-61421
送检材料：	肾脏组织			患者电话：	
临床诊断：	肾病综合征：膜性肾病？				

大体描述:

1：中性甲醛固定的条索状灰白色组织一条，长约（1.3cm）取一盒全用作光镜检查；
2：荧光保存液固定的条索状灰白色组织一条，长约（0.3cm）取一盒全用作免疫荧光检查；
3：中性戊二醛固定的条索状灰黄组织一条，长约（0.1cm）取一盒全用作电镜检查。

镜下描述（主要病变）：

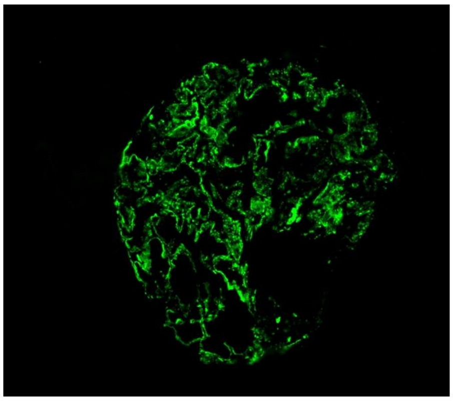

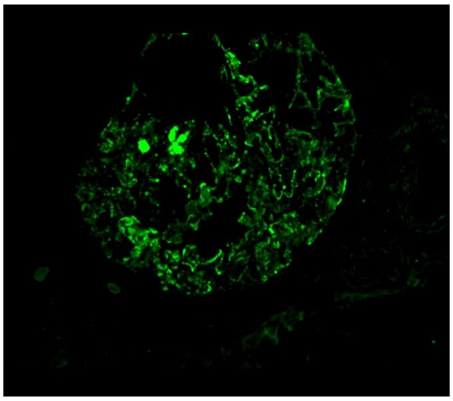

诊断意见:

免疫荧光：κ：+，λ：+，AA：阴性，Fib：阴性，ALB：肾小管可见重吸收的小滴，IgG1：阴性，IgG2：阴性，IgG3:+，IgG4：+++，PLA2R：+，HBsAg：阴性，HBcAg：阴性，HBeAg：阴性，C4d：球（+），THSD7a：阴性。

本检测仅对来样负责。如果对结果有疑义，请在收到结果后7个工作日内与我们联系，多谢合作！

报告医师：

查对技师：

报告日期：　2017-12-15 13:13:53

广东省人民医院-广州金域医学检验中心

本报告单经过电子签名认证

肾活检病理检查报告书

标本条码：	0137126028	医　院：	广东省人民医院-肾内科		
病人姓名：		科　室：	肾内科一区	病理号：	KB1737358
性　别：	男	房/床号：	29床	门诊/住院号	P771218
年　龄：	76　岁	接收日期：	2017-12-13 16:16:09	申请医生：	
项目名称：	常规肾脏病理检查			医生电话：	83827812-61421
送检材料：	肾脏组织			患者电话：	
临床诊断：	肾病综合征：膜性肾病？				

大体描述:

1：中性甲醛固定的条索状灰白色组织一条，长约（1.3cm）取一盒全用作光镜检查；
2：荧光保存液固定的条索状灰白色组织一条，长约（0.3cm）取一盒全用作免疫荧光检查；
3：中性戊二醛固定的条索状灰黄组织一条，长约（0.1cm）取一盒全用作电镜检查。

光镜描述

送检肾穿刺组织常规做HE、PAS、PASM、Masson染色，主要为肾皮质，可见21个肾小球，其中5个肾小球球性硬化，1个肾小球节段性硬化。
其余肾小球系膜细胞和基质中-重度弥漫性增生，以系膜基质增生更为明显，部分毛细血管腔狭窄，部分毛细血管襻血管瘤样扩张，少数肾小球系膜区呈结节状改变，可见K-W结节形成，基底膜显增厚，可见少量钉突样结构，上皮下可见嗜复红蛋白沉积，壁层上皮细胞无增生，未见新月体形成，部分肾小球球囊周可见纤维化。
肾小管上皮颗粒及空泡变性，可见少量蛋白管型，多灶状及片状萎缩（萎缩面积约60%），肾间质多灶状及片状炎症细胞浸润伴纤维化，小动脉管壁增厚，细动脉玻璃样变，管腔狭窄。

免疫荧光：　肾小球数 4 个

抗体种类:

IgG: +++　　IgM: +　　IgA: 阴性　　C3:　++　　C1q:　阴性

沉积部位: 弥漫、球性、毛细血管襻

沉积方式: 细颗粒状

病理诊断:

综合光镜、免疫荧光及电镜检查：
1、符合结节性糖尿病肾小球硬化症；
2、符合II期膜性肾病。

The manifestation of light microscope,immunofluorescence and electron microscope supported the diagnosis of nodular diabetic glomerulosclerosis and membranous nephropathy stage II.

本检测仅对来样负责。如果对结果有疑义，请在收到结果后7个工作日内与我们联系，多谢合作！　报告医师：

审核：　　报告日期：　2017-12-15 13:13:30

GZ17915158743727

广东省人民医院-广州金域医学检验中心

本报告单经过电子签名认证

肾活检病理检查报告书图片页

标本条码：	0137126028	医 院：	广东省人民医院-肾内科	病理号：	KB1737358
病人姓名：				门诊/住院号	P771218
性 别：	男	科 室：	肾内科一区	房/床号：	29床
年 龄：	76 岁	接收日期：	2017-12-13 16:16:09	申请医生：	
项目名称：	常规肾脏病理检查			医生电话：	83827812-61421
送检材料：	肾脏组织			患者电话：	
临床诊断：	肾病综合征：膜性肾病？				

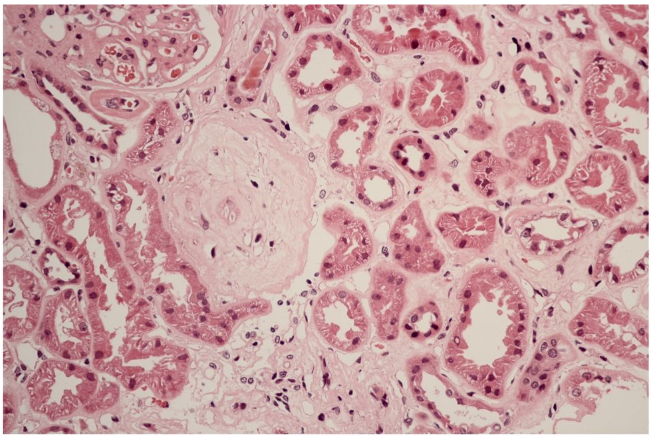

HE见硬化的肾小球

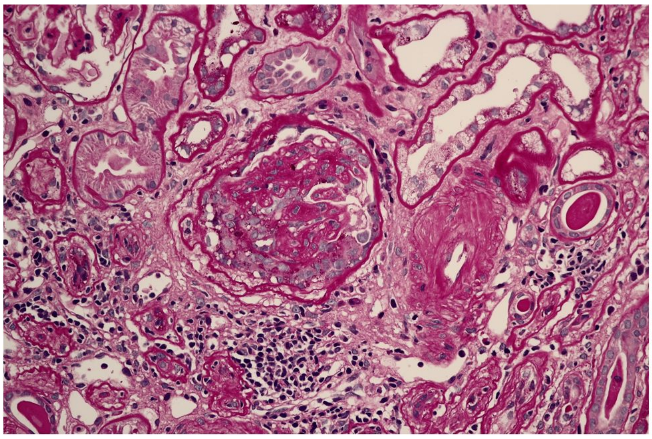

PAS见节段性硬化的肾小球

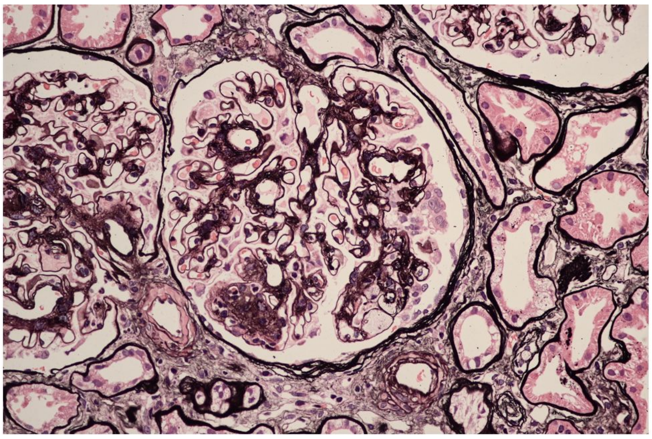

PASM见基底膜增厚，可见少量钉突样结构

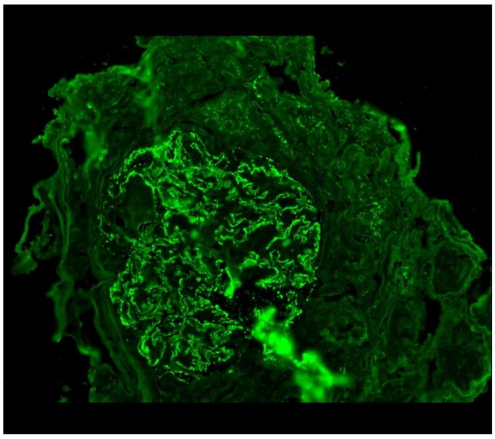

免疫荧光见IgG沉积

本检测为科研检测且仅对来样负责。如果对结果有疑义，请在收到结果后7个工作日内与我们联系，多谢合作！ 报告医师：

主检实验室：广州金域

GZ17915158743727

报告日期： 2017-12-15 13:13:30

广东省人民医院-广州金域医学检验中心

本报告单经过电子签名认证

肾活检病理检查电镜报告书

标本条码：	0137126028	医　院：	广东省人民医院-肾内科		
病人姓名：		科　室：	肾内科一区	病理号：	KB1737358
性　别：	男	房/床号：	29床	门诊/住院号	P771218
年　龄：	76　岁	接收日期：	2017-12-13 16:16:09	申请医生：	
项目名称：	普通透射电镜检查与诊断			医生电话：	83827812-61421
送检材料：	肾脏组织			患者电话：	
临床诊断：	肾病综合征：膜性肾病？			医院标识：	

大体描述:

1：中性甲醛固定的条索状灰白色组织一条，长约（1.3cm）取一盒全用作光镜检查；
2：荧光保存液固定的条索状灰白色组织一条，长约（0.3cm）取一盒全用作免疫荧光检查；
3：中性戊二醛固定的条索状灰黄组织一条，长约（0.1cm）取一盒全用作电镜检查。

电镜描述:

肾小球：镜下检测到1个肾小球。毛细血管内皮细胞明显空泡变性，个别管腔内可见红细胞聚集，无明显内皮细胞增生，部分毛细血管襻受压，部分管腔狭窄。肾小囊壁层增厚、分层，壁层细胞空泡变性，无明显增生。
基底膜：弥漫均质增厚，厚度达1200nm。
脏层上皮细胞：上皮细胞肿胀，空泡变性。足突弥漫融合。
上皮下、基底膜内多量电子致密物沉积。
系膜区：系膜细胞和基质增生，结节形成。
未见电子致密物沉积。
肾小管-间质：部分肾小管萎缩。肾间质胶原纤维增生伴淋巴、单个核细胞浸润。
肾间质血管：毛细血管管腔内见红细胞聚集，小动脉管壁增厚。

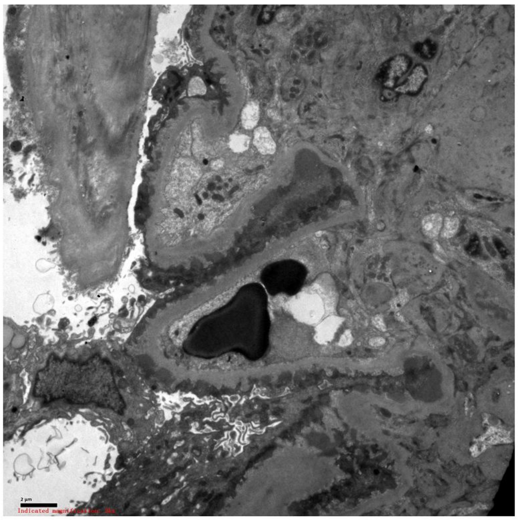

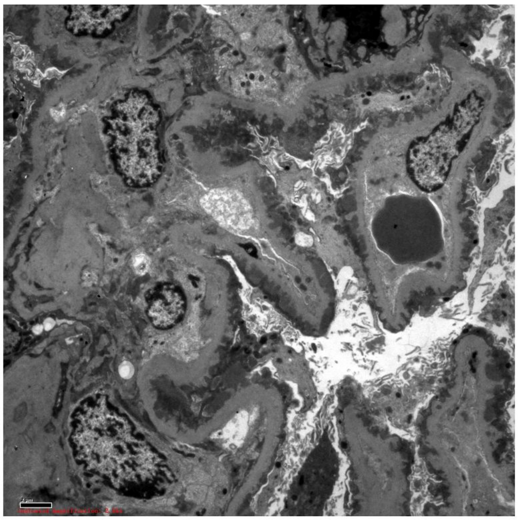

电镜诊断或印象:

综合光镜、免疫荧光及电镜检查：
1、符合结节性糖尿病肾小球硬化症；
2、符合Ⅱ期膜性肾病。

本检测仅对来样负责。如果对结果有疑义，请在收到结果后7个工作日内与我们联系，多谢合作！

报告医师：

审核：　　　报告日期：2017-12-15 13:12:59

GZ17915158664245

The manifestation of light microscope,immunofluorescence and electron microscope supported the diagnosis of nodular diabetic glomerulosclerosis and membranous nephropathy stage II.
