# Supplementary material for: The impact of coexisting diabetes mellitus on clinical outcomes in patients with idiopathic membranous nephropathy: a retrospective observational study
Source: BMC Nephrol. 2020 Jun 12;21:224. doi: 10.1186/s12882-020-01878-7 (PMC7291707; doi:10.1186/s12882-020-01878-7)
Supplement: Supplementary file 6 — Additional file 6. [file 12882_2020_1878_MOESM6_ESM.pdf]

广东省人民医院-广州金域医学检验中心

本报告单经过电子签名认证

病理诊断报告书

标本条码： 0128250206　　医　院： 广东省人民医院-肾内科
病人姓名：　　科　室： 肾内一区　　病理号： KB1700296
性　别： 女　　房/床号： 34床　　门诊/住院号: P710875
年　龄： 54　岁　　接收时间： 2016-12-29 22:58:13　　申请医生：
项目名称： 免疫荧光14项　　医生电话： 83827812-61421
送检材料： 肾脏组织　　患者电话： 15986926156
临床诊断： 肾病综合征 2型糖尿病肾病　2型糖尿病

大体描述:

1：中性甲醛固定的条索状灰白色组织二条，长约（0.7cm、0.9cm）取一盒全用作光镜检查；
2：荧光保存液固定的条索状灰白色组织一条，长约（0.3cm）取一盒全用作免疫荧光检查；
3：中性戊二醛固定的条索状灰黄组织一条，长约（0.15cm）取一盒全用作电镜检查。

镜下描述（主要病变）：

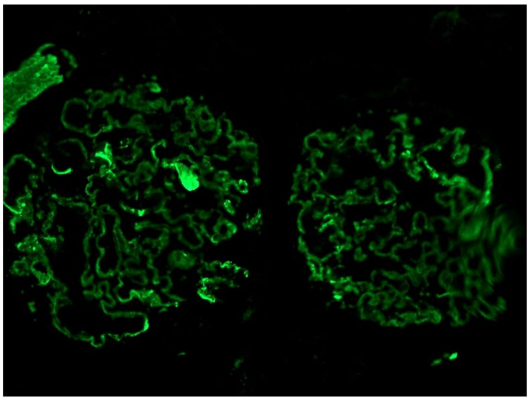

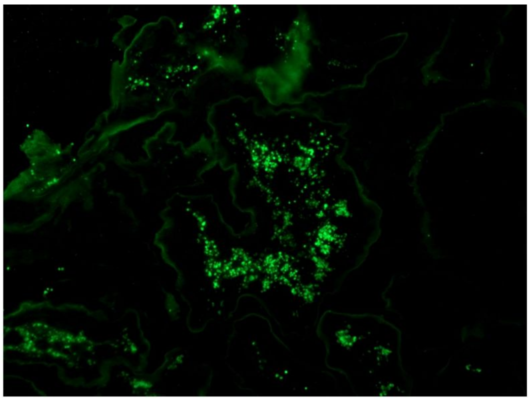

诊断意见:

免疫荧光：κ：+，λ：+，AA：阴性，Fib：阴性，ALB：肾小管可见重吸收的小滴，IgG1：++，IgG2：+，IgG3，阴性，IgG4：+++，PLA2R：+，HBsAg：阴性，HBcAg：阴性，HBeAg：阴性，C4d：阴性。

本检测仅对来样负责。如果对结果有疑义，请在收到结果后7个工作日内与我们联系，多谢合作！　　**报告医师：**

查对技师：　　**报告日期：** 2017-01-03 14:24:09

广东省人民医院-广州金域医学检验中心

本报告单经过电子签名认证

肾活检病理检查电镜报告书

标本条码：	0128250206	医　院：	广东省人民医院-肾内科		
病人姓名：		科　室：	肾内一区	病理号：	KB1700296
性　别：	女	房/床号：	34床	门诊/住院号	P710875
年　龄：	54　岁	接收日期：	2016-12-29 22:58:13	申请医生：	
项目名称：	普通透射电镜检查与诊断			医生电话：	83827812-61421
送检材料：	肾脏组织			患者电话：	15986926156
临床诊断：	肾病综合征 2型糖尿病肾病　2型糖尿病			医院标识：	

大体描述:

1：中性甲醛固定的条索状灰白色组织二条，长约（0.7cm、0.9cm）取一盒全用作光镜检查；
2：荧光保存液固定的条索状灰白色组织一条，长约（0.3cm）取一盒全用作免疫荧光检查；
3：中性戊二醛固定的条索状灰黄组织一条，长约（0.15cm）取一盒全用作电镜检查。

电镜描述:

肾小球：镜下检测到2个肾小球。毛细血管内皮细胞明显空泡变性，个别管腔内可见红细胞聚集，无明显内皮细胞增生，毛细血管襻开放。肾小囊壁层无明显增厚，壁层细胞空泡变性，无明显增生。
基底膜：均质增厚，厚度达2000nm，基底膜内见多量电子致密物沉积。
脏层上皮细胞：上皮细胞肿胀，空泡变性。足突弥漫融合，上皮下见电子致密物沉积。
系膜区：系膜细胞和基质增生，未见电子致密物沉积。
肾小管-间质：肾小管上皮细胞空泡变性，部分肾小管萎缩。肾间质炎症细胞浸润。
肾间质血管：个别毛细血管管腔内见红细胞聚集。小动脉管壁增厚。

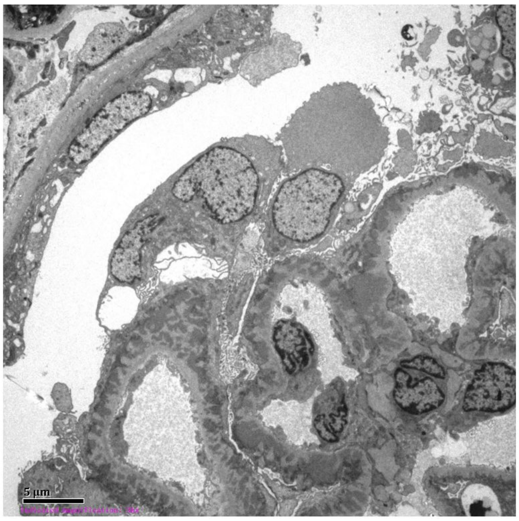

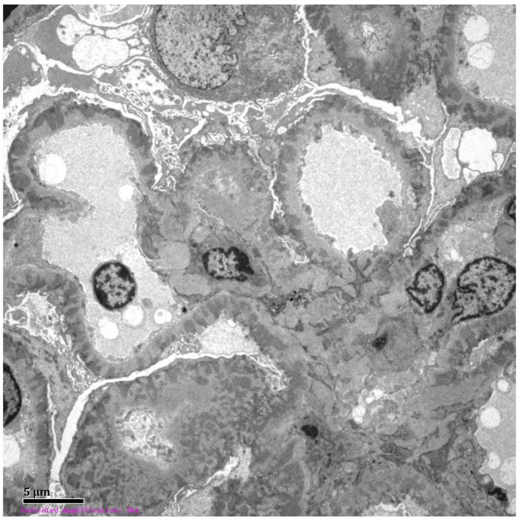

电镜诊断或印象:

综合光镜、免疫荧光及电镜检查：
符合III期膜性肾病伴轻-中度慢性肾小管-间质损伤。
注：此病例此次肾活检电镜下肾小球基底膜轻度均质增厚，不除外合并糖尿病肾病早期改变，请结合临床。

本检测仅对来样负责。如果对结果有疑义，请在收到结果后7个工作日内与我们联系，多谢合作！

报告医师：

审核：　　报告日期：2017-01-03 14:23:21

GZ17842559537304

The manifestation of light microscope,immunofluorescence and electron microscope supported the diagnosis of membranous nephropathy stage III and mild to moderate chronic tubulointerstitial injury.
Electron microscopy showed the mild and homogeneous thickening of basement membrane, which supported the diagnosis of the early stage of diabetic nephropathy.

广东省人民医院-广州金域医学检验中心

本报告单经过电子签名认证

肾活检病理检查报告书

标本条码：	0128250206	医　院：	广东省人民医院-肾内科		
病人姓名：		科　室：	肾内一区	病理号：	KB1700296
性　别：	女	房/床号：	34床	门诊/住院号	P710875
年　龄：	54　岁	接收日期：	2016-12-29 22:58:13	申请医生：	
项目名称：	常规肾脏病理检查			医生电话：	83827812-61421
送检材料：	肾脏组织			患者电话：	15986926156
临床诊断：	肾病综合征 2型糖尿病肾病　2型糖尿病				

大体描述：

1：中性甲醛固定的条索状灰白色组织二条，长约（0.7cm、0.9cm）取一盒全用作光镜检查；
2：荧光保存液固定的条索状灰白色组织一条，长约（0.3cm）取一盒全用作免疫荧光检查；
3：中性戊二醛固定的条索状灰黄组织一条，长约（0.15cm）取一盒全用作电镜检查。

光镜描述

送检肾穿刺组织常规做HE、PAS、PASM、Masson染色，主要为肾皮质，可见27个肾小球，未见肾小球球性硬化，其中1个肾小球节段性硬化。
其余肾小球系膜细胞和基质轻度弥漫性增生，毛细血管襻开放，外观显僵硬，基底膜增厚，可见较多钉突样结构，上皮下可见嗜复红蛋白沉积，未见纤维素样坏死，未见白金耳样结构，壁层上皮细胞无增生，未见新月体形成。
肾小管上皮细胞空泡及颗粒变性，可见少量蛋白管型，部分肾小管可见上皮的脱落，部分肾小管管腔缩小，基底膜增厚，肾间质灶状炎症细胞浸润伴轻度纤维化，小动脉管壁增厚，管腔狭窄。

免疫荧光：　肾小球数 8 个

抗体种类：

IgG：+++　　IgM：+　　IgA：阴性　　C3：　++　　C1q：　阴性

沉积部位：弥漫、球性、毛细血管襻

沉积方式：细颗粒状

病理诊断：

综合光镜、免疫荧光及电镜检查：
符合III期膜性肾病伴轻-中度慢性肾小管-间质损伤。
注：此病例此次肾活检电镜下肾小球基底膜轻度均质增厚，不除外合并糖尿病肾病早期改变，请结合临床。

本检测仅对来样负责。如果对结果有疑义，请在收到结果后7个工作日内与我们联系，多谢合作！

报告医师：

审核：　　　　报告日期：　2017-01-03 14:24:04

GZ17842560226684

The manifestation of light microscope,immunofluorescence and electron microscope supported the diagnosis of membranous nephropathy stage III and mild to moderate chronic tubulointerstitial injury.
Electron microscopy showed the mild and homogeneous thickening of basement membrane, which supported the diagnosis of the early stage of diabetic nephropathy.

广东省人民医院-广州金域医学检验中心

本报告单经过电子签名认证

肾活检病理检查报告书图片页

标本条码：	0128250206	医　院：	广东省人民医院-肾内科	病理号：	KB1700296
病人姓名：				门诊/住院号	P710875
性　别：	女	科　室：	肾内一区	房/床号：	34床
年　龄：	54　岁	接收日期：	2016-12-29 22:58:13	申请医生：	
项目名称：	常规肾脏病理检查			医生电话：	83827812-61421
送检材料：	肾脏组织			患者电话：	15986926156
临床诊断：	肾病综合征 2型糖尿病肾病　2型糖尿病				

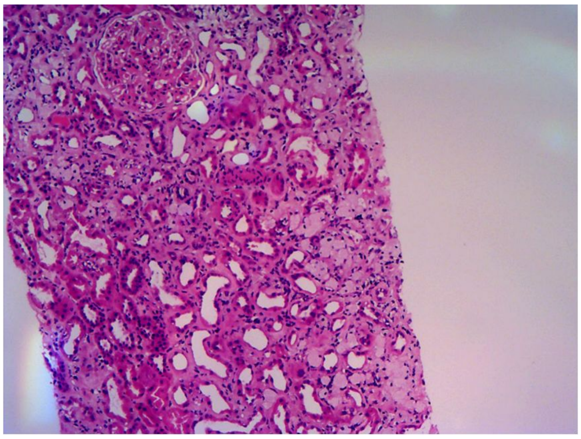

HE见肾间质灶状炎症细胞浸润伴轻度纤维化

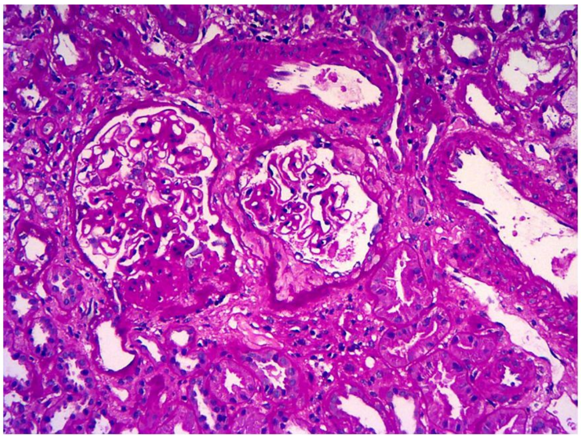

PAS见节段性硬化的肾小球

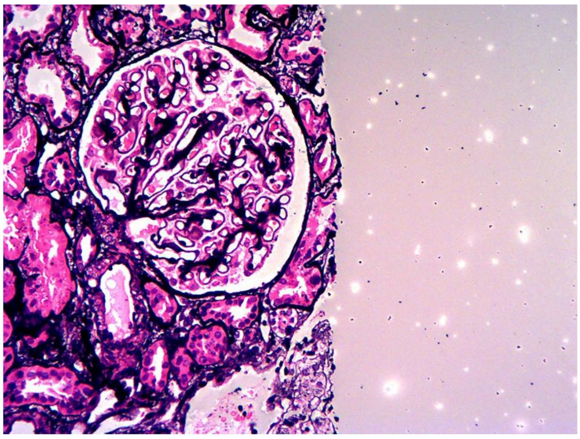

PASM见基底膜增厚，可见较多钉突样结构

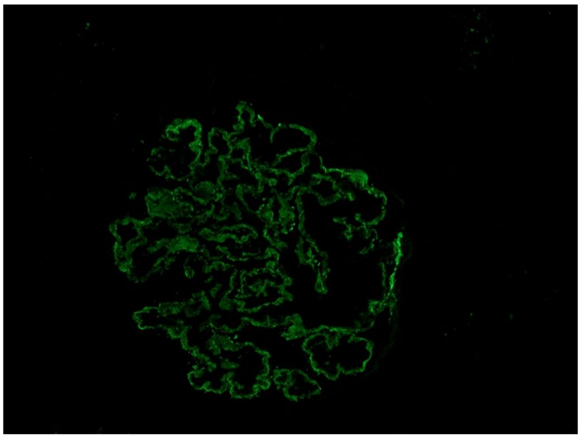

免疫荧光见IgG沉积

本检测为科研检测且仅对来样负责。如果对结果有疑义，请在收到结果后7个工作日内与我们联系，多谢合作！　报告医师：

主检实验室：肾脏常规岗位　GZ17842560226684　报告日期：　2017-01-03 14:24:04
